# Mathematical models of human mobility of relevance to malaria transmission in Africa

**DOI:** 10.1038/s41598-018-26023-1

**Published:** 2018-05-16

**Authors:** John M. Marshall, Sean L. Wu, Hector M. Sanchez C., Samson S. Kiware, Micky Ndhlovu, André Lin Ouédraogo, Mahamoudou B. Touré, Hugh J. Sturrock, Azra C. Ghani, Neil M. Ferguson

**Affiliations:** 1 MRC Centre for Outbreak Analysis and Modelling, Department of Infectious Disease Epidemiology, Imperial College London, London, UK; 2 Divisions of Biostatistics and Epidemiology, School of Public Health, University of California, Berkeley, California, USA; 3 Environmental Health and Ecological Sciences Thematic Group, Ifakara Health Institute, Dar es Salaam, Tanzania; 4 Chainama College of Health Sciences, Lusaka, Zambia; 5 Centre National de Recherche et de Formation sur le Paludisme, Ouagadougou, Burkina Faso; 6 Institute for Disease Modeling, Bellevue, Washington, USA; 7 Malaria Research and Training Center, University of Bamako, Bamako, Mali; 8 Malaria Elimination Initiative, Global Health Group, University of California, San Francisco, California, USA

## Abstract

As Africa-wide malaria prevalence declines, an understanding of human movement patterns is essential to inform how best to target interventions. We fitted movement models to trip data from surveys conducted at 3–5 sites throughout each of Mali, Burkina Faso, Zambia and Tanzania. Two models were compared in terms of their ability to predict the observed movement patterns – a gravity model, in which movement rates between pairs of locations increase with population size and decrease with distance, and a radiation model, in which travelers are cumulatively “absorbed” as they move outwards from their origin of travel. The gravity model provided a better fit to the data overall and for travel to large populations, while the radiation model provided a better fit for nearby populations. One strength of the data set was that trips could be categorized according to traveler group – namely, women traveling with children in all survey countries and youth workers in Mali. For gravity models fitted to data specific to these groups, youth workers were found to have a higher travel frequency to large population centers, and women traveling with children a lower frequency. These models may help predict the spatial transmission of malaria parasites and inform strategies to control their spread.

## Introduction

Increasing human mobility in Africa is creating highly favorable conditions for the persistence of diseases being targeted for elimination, such as malaria^1,2^, and for the faster spread of emerging pathogens, such as Ebola or Zika^3^. Significant funding is currently being invested in global malaria control and elimination^4^ and mathematical models are informing the most efficient use of these resources for reducing transmission^5^. As malaria transmission declines^6^, predictive models of human movement are needed to help inform how best to target interventions^7,8^. Empirical data on human movement are available for some locations^1,9–11^; however there are invariably biases inherent in all data sets and there are many locations for which data are not available^12^. Predictive movement models therefore provide an opportunity to extrapolate movement patterns to locations where data is biased or unavailable.

In recent years, two general classes of models have been proposed to describe patterns of human movement – gravity models^13,14^ and radiation models^15^. Gravity models assume movement rates between pairs of locations increase with the sizes of origin and destination populations and decrease with journey distance, akin to physical gravity. These models were used by Xia *et al*.^14^ to describe the spread of measles through populations in the UK prior to wide-scale vaccination programs. Variants have since been proposed in which the dependence on distance is described by a function that may depend on Euclidean distance, road distance, travel cost, travel time, or some combination of these metrics^16^. Radiation models take their inspiration from a simple particle diffusion model whereby travelers (the particles) are “absorbed” as they move outwards from their origin of travel, with the probability of absorption at a given radius being proportional to the population size within that radius^15^. These models were shown to apply well to US national and state-wide travel data originating from New York county^15^, and hence could be suitable for modeling the spatial spread of seasonal influenza and other infectious diseases in the US^17^. Variants have been proposed in which the spatial scale is varied, and directionality and other trip constraints are incorporated^18,19^.

Comparisons of model predictions to data suggest that these families of models describe commuting patterns in the US and UK reasonably well. However, they perform less well at capturing local movement patterns within large population centers, such as London or New York, where movement is goal-driven^14,15,18^. The gravity model provided a better overall fit to commuting data in the UK; however, the radiation model provided a better fit for small populations at large distances^18^. Wesolowski *et al*.^20^ compared the performance of gravity and radiation models in explaining movement patterns inferred from anonymous cell phone signal data in Kenya and suggested that travel in Kenya, and potentially in many parts of sub-Saharan Africa, may have unique features that are not well suited to description by these models. They noted the variable accessibility of rural destinations, in terms of cost, transport availability and road quality, and the rise of the mega-city, which is attractive beyond what would be expected due to population size alone. Furthermore, they noted that movement models tend to overestimate the number of destinations to which travelers move, and hence may overestimate the dispersal of disease.

Here, we explore the ability of gravity and radiation models to capture movement patterns from a survey conducted at 3–5 sites throughout each of four sub-Saharan African countries – Mali, Burkina Faso, Zambia and Tanzania^10^. A key feature of these data is that they were collected specifically to understand movement patterns of relevance to malaria transmission. Respondents were asked about trips for which they had spent at least one night away from home, which is required for malaria transmission since the main African malaria vectors, *Anopheles gambiae* and *Anopheles funestus*, bite at night. The surveys also asked questions about demography and trip details, which allowed us to classify trips made by key traveler groups – women traveling with children and youth workers – elucidated in previous analyses^10^. These traveler groups are of relevance to malaria transmission, since children are most likely to display clinical malaria incidence in high prevalence settings^21^, and movements of youth workers tend to correlate with seasonal rains and hence peak mosquito densities in the Sahel^22^. We therefore additionally explore the application of gravity and radiation models to describe movement patterns in these traveler groups specifically.

## Methods

### Data

We analyzed trip data from surveys carried out in four countries with ongoing malaria transmission – Mali and Burkina Faso in West Africa, and Zambia and Tanzania in East/Southern Africa. The data are described elsewhere^10^. In brief, participants were asked a series of questions about the last up to three trips undertaken in the previous year, restricted to those for which they spent at least one night away from home. Information collected included trip details (purpose, duration, month of departure and number of accompanying children) and basic demographic information (age, gender and number of children under the age of five). The surveys were conducted at 3–5 sites per country, chosen according to a judgment/convenience sample. Models fitted to these data therefore reflect the collection of sites and may only serve as proxies for the countries at large. Travel within the ward, commune or city of origin was not recorded. Study participants were interviewed in Mali during the rainy season of September/October 2010 and the dry season of March 2011, in Burkina Faso during the rainy season of July 2011, in Zambia during the cool dry season of July/August 2012, and in Tanzania during the long rainy season of March 2013.

For the purpose of model fitting, only trips for which both the origin and destination were resolved at the administrative level of commune in Mali and Burkina Faso and at the administrative level of ward in Zambia and Tanzania were retained. This represented 96.4% of trips in Mali, 99.2% of trips in Burkina Faso, 77.0% of trips in Zambia and 98.9% of trips in Tanzania. Trips were assigned to traveler groups based on a cluster analysis accounting demographic and trip details^10^. For Mali and Burkina Faso, the population size within each commune was estimated using WorldPop population estimates^23^ and the coordinates for each commune were taken as the population-weighted centroids. For Zambia and Tanzania, the lists of wards used in the surveys and their corresponding population sizes were taken from recent censuses^24,25^. These lists of wards did not correspond to any one set of shape files and so, where possible, population-weighted centroids were estimated using WorldPop^23^, and otherwise coordinates were taken from GADM shape files (www.gadm.org) and the Stanford Digital Repository (https://purl.stanford.edu/rn812zx7730). The collated data and estimates are provided in Supplementary File 1.

Our surveys did not collect information on individuals who had not traveled in the last year, and so to determine whether origin population size had a significant influence on movement frequency, we used equivalent data from national Demographic and Health Surveys (DHS) for each of the survey countries^26–29^. Geocoded responses to question V167, which measured the number of overnight trips taken by respondents in the last year, were linked to communes/wards having the nearest centroid and used to calculate the proportion of male and female respondents who didn’t travel and the mean number of trips taken by male and female respondents, with weightings to account for differences in demographic sampling rates. These summary statistics were then plotted against origin population size for each survey country. Interestingly, there was no suggestion of a relationship between origin population size and travel frequency for either males or female respondents (Supplementary Figure 1), allowing us to explore the application of gravity and radiation models conditional upon the location of origin.

### Gravity models

A gravity model was fitted to the trips recorded for each country. Model fitting was carried out: (a) on the full set of trips for each country, (b) on trips subdivided according to traveler group – (i) women traveling with children (for all survey countries), and (ii) youth workers (for Mali) – and (c) on the full set of trips for all countries simultaneously. The gravity model was formulated conditional upon the origin, *i*, such that the probability of a trip being to destination *j* is proportional to the population size at *j*, *N*_*j*_, raised to a power, *τ*, and proportional to a distance kernel, *k*(*d*_*i*,*j*_), that is a function of the distance between the two locations:

1 $$P(j|i)\propto {N}_{j}^{\tau }k({d}_{i,j}).$$

Analyses were conducted at the national scale. Lognormal, power law and exponential distance kernels were considered, however the power law kernel, parameterized by a scale parameter, *ρ*, and a power parameter, *α*, provided the best fit:

2 $$k({d}_{i,j})={(1+\frac{{d}_{i,j}}{\rho })}^{-\alpha }.$$

Models both with and without a power, *τ*, on the destination population size were considered. Simplified gravity models conditional upon the origin of travel were considered because: (i) the data for each country were restricted to 3–5 origins, and (ii) data from national DHS surveys for each of the survey countries suggested no apparent relationship between travel frequency and origin population size (Supplementary Figure 1).

### Radiation models

Several variants of the radiation model were also fitted to the trips recorded for each country, to trips subdivided according to traveler group, and to trips for all countries simultaneously. In the basic radiation model, given an origin *i*, the probability of a journey ending at a location greater than a distance *r* away, P_*i*_(*d* > *r*), is inversely proportional to the population size living within a ring of radius *r* around *i*. Mathematically, this may be formulated as,

3 $${{\rm{P}}}_{i}(d > r)\propto \frac{1}{{s}_{i}(r)},$$

where *s*_*i*_(*r*) represents the total population size living within a ring of radius *r* centered around origin *i*.

The basic radiation model has no free parameters. However, because we were only considering journeys involving an overnight stay, we modified the model to generate less local journey distance distributions. We considered two variants of this model. In the first (model A), the origin population size, *N*_*i*_, is scaled by a factor *u*:

4 $${{\rm{P}}}_{i}(d > r)\propto \frac{1}{({s}_{i}(r)-{N}_{i})/u+{N}_{i}},$$

The second model variant (model B) effectively adds a fixed value *v* to the origin population:

5 $${{\rm{P}}}_{i}(d > r)\propto \frac{1}{{s}_{i}(r)+v}.$$

Parameters *u* and *v* were estimated in the model fitting process.

### Model fitting

Model parameters were estimated using a Markov chain Monte Carlo (MCMC) algorithm. The log likelihood of the data, *D*, given model parameters, $\theta =\{\alpha ,\rho ,\tau \}$ for the gravity model and *θ* = *μ* or *θ* = *v* for the radiation model, was calculated using the expected distribution of destinations for each origin given the movement model and chosen parameter values and summing the log likelihood over trips recorded at the administrative level of ward and/or commune, i.e.:

6 $$\mathrm{log}\,L(\theta )=\sum _{k=1}^{K}P({j}_{k}|{i}_{k},\theta ).$$

Here, *K* represents the number of recorded trips resolved at the level of ward or commune and *i*_*k*_ and *j*_*k*_ are the origin and destination for the *k*th trip, respectively. The deviance information criterion (DIC) was used as a measure for model selection^30^, defined as:

7 $${\rm{DIC}}=-\,2\,\mathrm{log}\,L(\bar{\theta })+2{p}_{D}.$$

Here, the first term represents the deviance, *D*(*θ*), defined as −2 times the log likelihood of the model, with model parameters equal to their means from the MCMC chain at equilibrium, and *p*_*D*_ represents the effective number of parameters, calculated here as the mean deviance of the MCMC chain at equilibrium minus the deviance with model parameters equal to their means from the MCMC chain at equilibrium, i.e. ${p}_{D}=\bar{D}(\theta )-D(\bar{\theta }).$ The relative prediction error of trip frequencies (defined as |1 − (*observed*/*predicted*)|) was used as a measure to present model fit graphically.

### Data availability

The data sets generated and analyzed in this study are available in Supplementary File 1.

## Results

**Figure 1:**
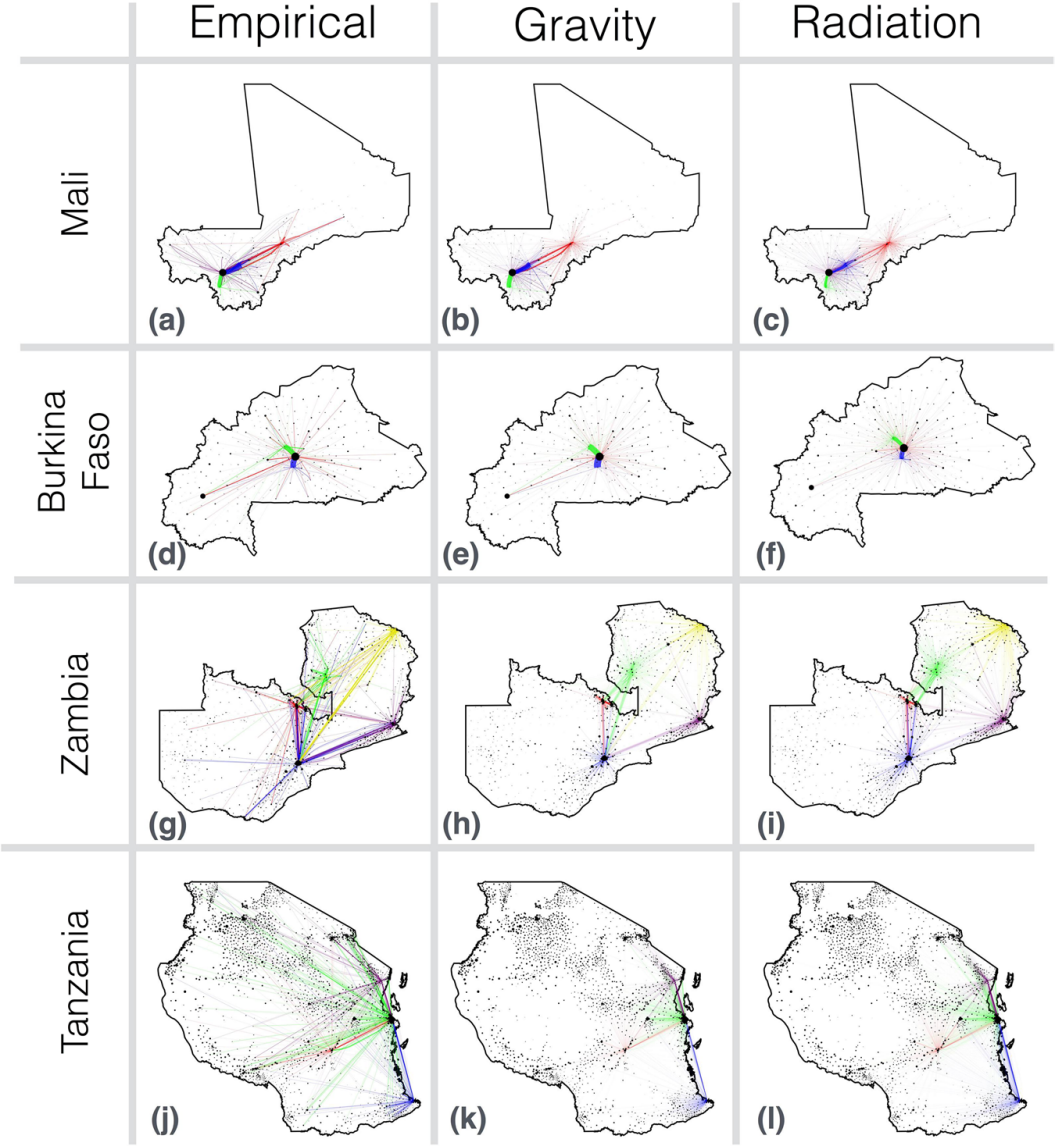
Empirical and model-predicted travel frequencies for each survey country. Model predictions are for the gravity model with the destination population size raised to a power, *τ*, and radiation model B fitted to data for each country individually. Each dot represents a commune or ward, the radius of which is a monotonically increasing function of its population size. Each line represents travel frequency between communes/wards, the width of which is proportional to travel frequency. Maps were generated using Mathematica version 11 (https://www.wolfram.com/mathematica/) with political boundaries obtained from Wolfram’s Data Repository (https://datarepository.wolframcloud.com/). The survey was conducted at 3–5 sites in each country, and trips are color-coded according to the survey location. In Mali (panels a–c), purple trips originate in Bamako, the capital city and largest urban center, green trips originate in the fishing village of Baya, blue trips originate in the farming villages of Barouéli and Boidié, and red trips originate in Mopti and Fatoma, a commercial center and village respectively. In Burkina Faso (panels d–f), red trips originate in Ouagadougou, the capital and largest city, blue trips originate in Sapone, an agricultural village, and green trips originate in Boussé, a local center of agriculture and trade. In Zambia (panels g–i), blue trips originate in Lusaka, the capital and largest city, green trips originate in Samfya, a central fishing town, red trips originate in Kitwe, an urban trading town in the Copperbelt, yellow trips originate in Nakonde, a town in the north-east bordering Tanzania, and purple trips originate in Chipata, a rural town in the east bordering Malawi. In Tanzania (panels j–l), green trips originate in Dar es Salaam, the capital and largest city, red trips originate in Ifakara, a small rural town on the edge of the Kilombero valley, purple trips originate in Muheza, a small rural town near the border with Kenya, and blue trips originate in Mtwara, an agricultural town with a growing mining industry near the border with Mozambique.

**Figure 2:**
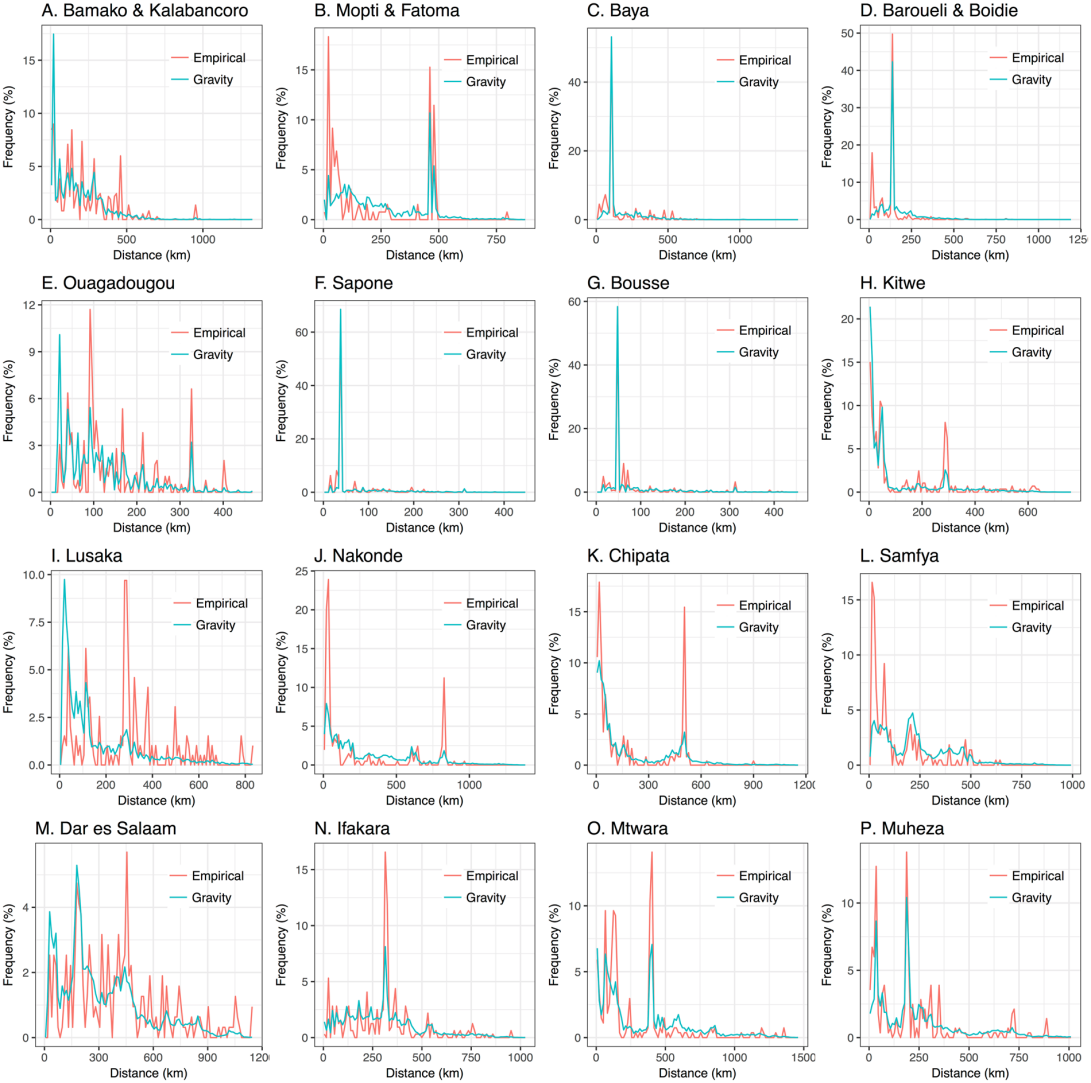
Empirical and model-predicted distance distributions for gravity model fitted to individual countries. Predicted travel frequencies are from the gravity model with the destination population size raised to a power, *τ*, and parameter values in Table 1. Distance distributions are shown for trips beginning at survey sites in Mali (panels A–D), Burkina Faso (panels E–G), Zambia (panels H–L) and Tanzania (panels M–P).

**Figure 3:**
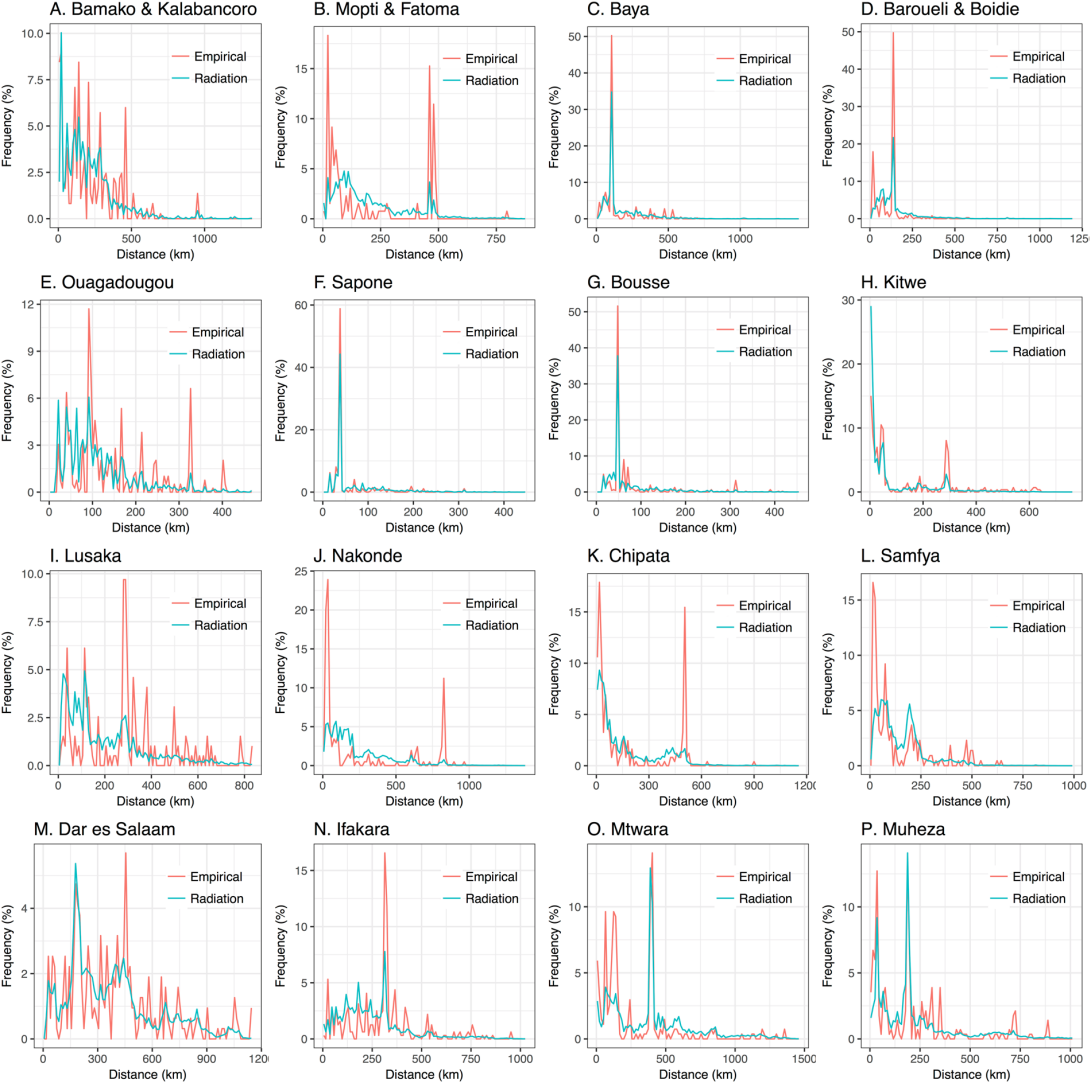
Empirical and model-predicted distance distributions for radiation model fitted to individual countries. Predicted travel frequencies are from radiation model B and parameter values in Table 1. Distance distributions are shown for trips beginning at survey sites in Mali (panels A–D), Burkina Faso (panels E–G), Zambia (panels H–L) and Tanzania (panels M–P).

The best-fitting movement model for the complete set of origins and destinations for each country, and for all countries combined, was a gravity model with a power law distance kernel and a power on the destination population size (Table 1). The estimated power on the destination population size was significantly larger than one for Mali and Burkina Faso (95% credible intervals (CrIs) of 1.21–1.27 and 1.30–1.38, respectively), and significantly less than one for Zambia and Tanzania (95% CrIs of 0.83–97 and 0.79–0.94, respectively). This can be interpreted as large population centers being more attractive in Mali and Burkina Faso than in Zambia and Tanzania. Which radiation model variant fitted better depended on country – model A (equation 4) was preferred for Mali, while model B (equation 5) was preferred for Burkina Faso, Zambia, Tanzania and for all countries combined. Model predictions for the best fitting gravity model and radiation model B are graphically depicted alongside the empirical survey data in Figs 1–3.

**Table 1:** Movement model parameters (with 95% credible intervals) for gravity and radiation models fitted to origin-destination pairs in all survey countries.

Model:	Parameters:	Mali:	Burkina Faso:	Zambia:	Tanzania:	All countries:
Gravity model (Equations 1–2) with *τ*	*α*	2.00 (1.62–2.58)	1.27 (1.18–1.38)	1.70 (1.54–1.88)	3.62 (2.78–5.16)	1.91 (1.78–2.06)
	log(*ρ*)	4.98 (4.52–5.47)	0.54 (0.02–1.80)	3.65 (3.31–3.97)	5.90 (5.41–6.47)	4.29 (4.09–4.48)
	*τ*	1.239 (1.211–1.267)	1.342 (1.304–1.381)	0.91 (0.83–0.97)	0.86 (0.79–0.94)	1.22 (1.20–1.24)
	DIC	12,027	8,444.4	14,593	16,762	52,206
Gravity model (Equations 1–2) without *τ*	*α*	2.12 (1.78–2.63)	1.70 (1.48–1.99)	1.74 (1.58–1.93)	3.43 (2.71–4.64)	1.84 (1.73–1.98)
	log(*ρ*)	4.83 (4.43–5.25)	2.64 (1.47–3.32)	3.75 (3.43–4.05)	5.79 (5.33–6.29)	4.10 (3.93–4.29)
	DIC	12,305	8,757.5	14,598	16,771	52,642
Radiation model A (Equation 4)	*u*	66.2 (58.8–74.7)	34.2 (29.9–39.1)	37.8 (33.7–42.8)	210 (184–240)	68.3 (64.0–72.9)
	DIC	12,149	8,653	14,719	16,931	52,860
Radiation model B (Equation 5)	*v*	2.67 (2.37–3.00) × 10^6^	1.80 (1.58–2.06) × 10^6^	6.61 (5.80–7.60) × 10^5^	3.66 (3.24–4.19) × 10^6^	1.96 (1.84–2.10) × 10^6^
	DIC	12,222	8,649	14,657	16,886	52,761

The performance of both models at capturing travel to the capital city varied as, for the gravity model, the “gravitational pull”, and for the radiation model, the absorption of the capital city, is balanced in the fitting alongside other major population centers (Table 2). For Mali, the gravity model underestimated travel to the capital (Bamako) from Barouéli/Boidié and Mopti/Fatoma; but captured it well for Baya. The radiation model, on the other hand, underestimated travel to Bamako from all other survey sites, especially from Barouéli/Boidié and Mopti/Fatoma. For Burkina Faso, the gravity model overestimated travel to the capital (Ouagadougou), while the radiation model underestimated it. For Zambia, travel to the capital (Lusaka) was greatly underestimated by both the gravity and radiation models from all survey sites except for Samfya, from which the radiation model slightly underestimated travel while the gravity model provided a good estimate. Empirically, low frequency movement from Samfya to Lusaka could have been due to the Democratic Republic of Congo having territory between these two locations. For Tanzania, the radiation model provided a good prediction of travel to the capital (Dar es Salaam) from Mtwara and Muheza, while underestimating travel from Ifakara. The gravity model slightly underestimated travel to Dar es Salaam from Mtwara and Ifakara; but provided a good prediction from Muheza.

**Table 2:** Observed versus predicted trip frequencies to capital cities for each survey country. Here, observed trip frequencies are the number of observed trips to the capital city divided by the total number of observed trips.

Trip origin and destination:	No. observed trips to capital city/total no. observed trips:	Observed trip frequency (95% CI):	Expected trip frequency (gravity model):	Expected trip frequency (radiation model):
Mopti & Fatoma – Bamako	32/131	0.24 (0.18–0.32)	0.140	0.042
Baya – Bamako	187/400	0.47 (0.42–0.52)	0.495	0.283
Barouéli & Boidié – Bamako	183/380	0.48 (0.43–0.53)	0.369	0.170
Sapone – Ouagadougou	157/272	0.58 (0.52–0.63)	0.675	0.429
Bousse – Ouagadougou	240/467	0.51 (0.47–0.56)	0.578	0.362
Kitwe – Lusaka	44/286	0.15 (0.12–0.20)	0.040	0.040
Nakonde – Lusaka	32/205	0.16 (0.11–0.21)	0.034	0.014
Chipata – Lusaka	46/246	0.19 (0.14–0.24)	0.039	0.022
Samfya – Lusaka	13/217	0.060 (0.035–0.100)	0.048	0.014
Ifakara – Dar es Salaam	96/320	0.30 (0.25–0.35)	0.105	0.100
Mtwara – Dar es Salaam	69/270	0.26 (0.21–0.31)	0.130	0.234
Muheza – Dar es Salaam	59/283	0.21 (0.17–0.26)	0.188	0.238

Expected trip frequencies are the equivalent quantity predicted by radiation model B and the gravity model with the destination population size raised to a power, *τ*, as parameterized in Table 1. 95% confidence intervals for observed trips assume a binomial distribution.

Another important factor in determining model fit is accurately capturing travel to nearby locations. These destinations also have a strong gravitational attractiveness or absorption potential; however, since we are only interested in overnight trips for malaria transmission, travelers may return home rather than sleeping nearby and hence the attractiveness of nearby locations may be lower. Figure 2 suggests that the gravity model tends to overestimate travel from capital cities to locations within their vicinity (expected – observed travel frequencies are significantly greater than 0 for destinations within 200 km of Bamako (t-test, p-value = 0.035), Lusaka (p-value < 10^−5^) and Dar es Salaam (p-value = 0.026)). Figure 3 suggests that the radiation model provides a closer fit for travel originating at a capital city (expected – observed travel frequency is only significantly different from 0 for destinations within 200 km of Lusaka (t-test, p-value < 10^−5^)). Travel within 20 km of the capital city of Mali, Bamako, is substantially underestimated by both models (t-test, p-value = 0.041 for the radiation model and 0.0009 for the gravity model); however, the fit to the Mali survey data is challenged by the high levels of movement within the vicinity of Mopti/Fatoma and Barouéli/Boidié but not from other origins. Similarly, for the Zambia survey data, frequent nearby movement recorded for all survey sites except the capital city, Lusaka, was hard to reconcile with either model. Rather, the radiation model provides a superior fit for travel within the vicinity of Lusaka (error variance for the radiation model is less than that for the gravity model for destinations within 200 km of Lusaka (F-test, p-value = 0.00002)), while the gravity model provides a superior fit for travel within the vicinity of another city – Nakonde (F-test, p-value = 0.043). Both models generally provide a good fit for travel within the vicinity of Burkina Faso and Tanzania survey sites (expected – observed travel frequencies are not significantly different from 0 for destinations within 200 km of all three survey sites in Burkina Faso and for Ifakara, Mtwara and Muheza in Tanzania (t-tests, p-values > 0.05)).

**Figure 4:**
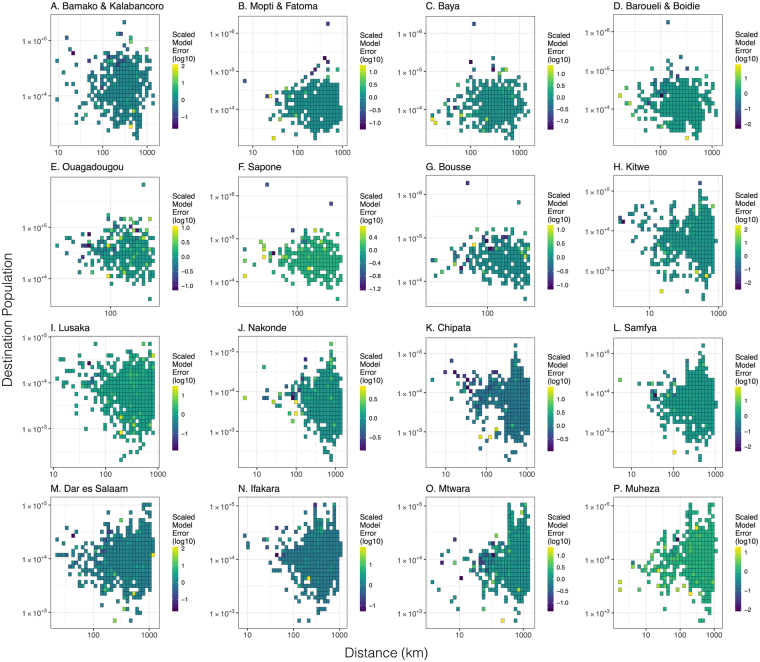
Relative prediction error for gravity model fitted to individual countries. Relative prediction error (absolute value of the difference between empirical and predicted travel frequency divided by the predicted travel frequency) versus destination population size and trip distance for trips beginning at survey sites in Mali (panels A–D), Burkina Faso (panels E–G), Zambia (panels H–L) and Tanzania (panels M–P). Predicted travel frequencies are from the gravity model fitted to individual countries with the destination population size raised to a power, *τ*, and parameter values in Table 1. Grid cells represent the average scaled model error for destinations falling within the corresponding range of destination population sizes and trip distances.

**Figure 5:**
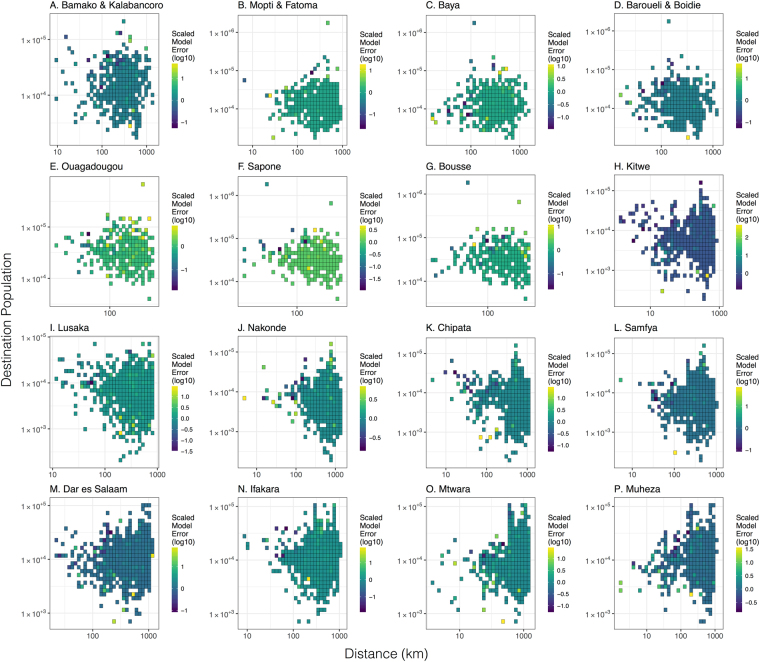
Relative prediction error for radiation model fitted to individual countries. Relative prediction error versus destination population size and trip distance for trips beginning at survey sites in Mali (panels A–D), Burkina Faso (panels E–G), Zambia (panels H–L) and Tanzania (panels M–P). Predicted travel frequencies are from radiation model B fitted to individual countries with parameter values in Table 1. Grid cells represent the average scaled model error for destinations falling within the corresponding range of destination population sizes and trip distances.

Plots of relative prediction error (Figs 4 and 5) show that both the gravity and radiation models give reasonable estimates of travel frequency across the range of intermediate distances and population sizes, with large errors often seen at either end of the trip distance and population size spectra, where recorded numbers of journeys are low and statistical noise is higher. For instance, for the gravity model applied to trips originating in Bamako/Kalabancoro, some of the largest scaled errors are seen for trips to distant, large populations (e.g. Goumera, Fanga and Tenenkou), whereas for the gravity model applied to trips originating in Mopti/Fatoma, some of the largest relative errors are for trips to nearby, large populations (e.g. Fatoma and Bassirou). Whilst it does not apply universally, there is a tendency for the radiation model to provide a better fit than the gravity model for trips to nearby populations. This is visually apparent in the distance distribution plots in Figs 2 and 3 and is evidenced by the error variance for the radiation model being less than that for that for the gravity model for trips within 200 km of nine out of 16 survey sites. This difference in error variance is statistically significant for trips within 200 km of Sapone, Burkina Faso (F-test, p-value = 0.0069) and Lusaka, Zambia (F-test, p-value = 0.00002). Conversely, there is a tendency for the gravity model to provide a better fit than the radiation model for trips to large populations. This is also visually apparent in the distance distribution plots in Figs 2 and 3 and is evidenced by the error variance for the gravity model being less than that for the radiation model for trips to the most populous 25% of destinations for each country for 10 out of 16 survey sites. This difference in error variance is statistically significant for trips from Mopti and Barouéli in Mali (F-test, p-values = 0.00017 and <10^−5^, respectively), and Kitwe and Nakonde in Zambia (F-test, p-values = 0.019 and 9.3 × 10^−5^, respectively).

Based on the results of the countrywide analysis, we fitted the gravity model with a power on the destination population size and radiation model B to the country data sets stratified by traveler group: (i) women traveling with children for each and all counties, and (ii) youth workers for Mali (Table 3). As previously illustrated in these data^10^, the “women with children” traveler group reported shorter distance trips for all survey countries. For the gravity model in this case, we found that the power, *τ*, of the destination population is smaller for the “women with children” cluster and larger for the “youth worker” cluster (Tables 1 and 3). This could indicate a smaller “gravitational pull” of large population centers for the “women with children” traveler group and a larger “gravitational pull” for the youth worker traveler group. This is particularly apparent for the “women with children” cluster in the Tanzania data set, for which travel frequencies to the capital and largest city, Dar es Salaam, are significantly smaller from Ifakara (0.12, 95% confidence interval (CI): 0.06–0.23 for women with children c.f. 0.33, 95% CI 0.28–0.39 for other travelers), Mtwara (0.14, 95% CI: 0.08–0.24 for women with children c.f. 0.30, 95% CI 0.24–0.37 for other travelers) and Muheza (0.05, 95% CI: 0.02–0.12 for women with children c.f. 0.28, 95% CI 0.22–0.34 for other travelers) (Fig. 2 and Supplementary Figure 2). For the radiation model applied to traveler groups, we found that the parameter, *v*, is smaller for the “women with children” cluster and larger for the “youth worker” cluster for all country data sets (Tables 1 and 3). This could indicate higher “absorption” of nearby populations for the “women with children” traveler group and smaller “absorption” of nearby populations for the youth worker traveler group. Both the gravity and radiation models provide similar overall trends for traveler groups as seen for all trips, as indicated by the distance frequency plots for both clusters (Supplementary Figures 2 and 4) and all trips (Figs 2 and 3). However, larger fluctuations are seen around model predictions for traveler groups, reflected in the corresponding plots of relative predictive error (Figs 4 and 5, Supplementary Figures 3 and 5), as a consequence of the smaller sample sizes in partitioned data.

**Table 3:** Gravity model parameters (with 95% credible intervals) for models fitted to origin-destination pairs stratified by traveler group (W&C: women traveling with children; YW: youth workers) in all survey countries.

Model:	Parameters:	Mali (W&C):	Mali (YW):	Burkina Faso (W&C):	Zambia (W&C):	Tanzania (W&C):	All countries (W&C):
Gravity model with *τ*	*α*	1.76 (1.33–2.55)	23.6 (2.8–70.6)	1.72 (1.54–2.07)	1.86 (1.60–2.19)	2.32 (1.89–2.92)	2.01 (1.83–2.23)
	log(*ρ*)	3.97 (3.05–4.87)	8.90 (6.46–9.96)	1.03 (0.05–2.75)	3.40 (2.85–3.92)	4.22 (3.59–4.85)	3.68 (3.37–4.00)
	*τ*	1.20 (1.14–1.26)	1.26 (1.21–1.31)	1.21 (1.14–1.28)	0.76 (0.62–0.90)	0.59 (0.40–0.77)	1.12 (1.08–1.16)
	DIC	2,472	4,390	2,496	4,010	3,591	12,679
Radiation model B	*v*	1.95 (1.52–2.52) × 10^6^	4.99 (3.99–6.30) × 10^6^	1.13 (0.88–1.45) × 10^6^	3.55 (2.76–4.50) × 10^5^	7.66 (5.84–9.99) × 10^5^	8.56 (7.52–9.74) × 10^5^
	DIC	2,518	4,486	2,508	4,027	3,618	12,758

The gravity and radiation models fitted to all trips in all countries simultaneously provide a reasonable fit, despite representing a compromise between the patterns observed in each country (Supplementary Figures 6 and 7 for the gravity model and Supplementary Figures 8 and 9 for the radiation model). For the gravity model, the all-country fit predictions for Mali are very similar to the Mali-only predictions (Figs 2 and 3), as expected given the similarity of parameter estimates (Table 1). For Burkina Faso, the all-country predictions further underestimate travel from Ouagadougou to large population centers and slightly underestimate travel from Sapone and Bousse to Ouagadougou due to a reduced *τ* parameter; however, the fit is generally comparable (Fig. 2). For Zambia and Tanzania, the increased *τ* parameter from the all-country fit results in a higher travel frequency to large, distant populations and a smaller travel frequency to small, nearby populations. This results in a slightly better fit in some cases (e.g. error variance within the vicinity of Dar es Salaam is slightly reduced and trip frequency from Kitwe to Lusaka is closer to the observed value) and a slightly worse fit in others (e.g. error variance within the vicinity of Lusaka is slightly increased). For the radiation model, the all-country fit predictions for Mali and Burkina Faso are very similar to the Mali-only and Burkina Faso-only predictions (Figs 4 and 5), as expected given the similarity of parameter estimates (Table 1). For Zambia, the increased *v* parameter from the all-country fit results in slightly less travel to nearby populations, and for Tanzania, the decreased *v* parameter from the all-country fit results in slightly more travel to nearby populations.

## Discussion

We fitted human movement models to trip data from a survey conducted in Mali, Burkina Faso, Zambia and Tanzania^10^. Two benefits of these data were that: (i) only overnight trips were recorded – i.e. trips of relevance to malaria transmission – and (ii) demographic and trip details were recorded, allowing trips to be categorized according to traveler group – namely, women traveling with children in all survey countries and youth workers in Mali. Two models were compared in terms of their ability to predict the observed movement patterns: (i) a gravity model, in which movement rates between pairs of locations increase with population size and decrease with the distance^14^, and (ii) a radiation model, in which travelers are cumulatively “absorbed” as they move outwards from their origin of travel^15^. The gravity model provided the best fit to the data overall, as measured by the likelihood of the data under each model; however, neither model was uniformly superior in its predictive ability. In general, the gravity model provided a better fit for travel to large populations, while the radiation model provided a better fit for nearby populations. Significantly different model parameters were obtained for two traveler groups compared to the population as a whole, with youth workers being more attracted to large population centers, and women traveling with children being less attracted (Table 3).

A number of approaches could be explored to improve the quality of the model fit to our data. For instance, since we are interested in overnight trips, and these may be less frequent to nearby locations as travelers are able to sleep at home, then we could consider down-weighting the distance kernel of the gravity model for shorter distances to compensate for this. The down-weighting could also be specific to the origin, since limited travel was observed within the vicinity of Bamako in Mali and Lusaka in Zambia; but significant travel was observed within the vicinity of Mopti in Mali and Nakonde in Zambia. It is difficult to generalize to the continent since our survey only covers four countries; however, at least for these four countries, down-weighting the distance kernel for short travel distances seems appropriate at least for capital cities. Another potential approach, given the impressive fit of the radiation model for nearby locations, would be to apply a gravity-radiation-hybrid model for movement prediction, in which the radiation model is applied for trips up to a certain radius and the gravity model is applied beyond this radius, with the critical radius being a free parameter to be determined through the model fitting process.

Two other issues that could be further explored, both mentioned by Wesolowski *et al*.^20^ include: (i) the “gravitational pull” of the mega-city, and (ii) the use of alternative distance metrics. Capital cities in many African countries represent unique sources of work, trade, healthcare and resources that may not be available elsewhere^2,22^. Furthermore, as a growing number of people migrate to these urban centers, subsequent family-related travel also increases^31^. The gravity model, with travel frequency being proportional to the destination population size raised to a variable power, is particularly well suited to accommodating the exceptional pull of African mega-cities; however, a consistently good fit is difficult if not impossible to obtain as the gravity model over-predicts travel to the capital city in some cases and under-predicts in others. Interestingly, travel to the capital city is generally well predicted by the gravity model applied to Mali and Burkina Faso; but is under-predicted by the same model applied to multiple survey sites in Zambia and Tanzania. Up-scaling of travel to the capital city could be considered in these cases; but the generalizability of this up-scaling factor is unclear.

Secondly, a major factor in determining travel frequency in Africa is road quality, availability of public transport and travel cost. These aspects of accessibility would potentially be better accommodated using road distance, travel cost or travel time in the place of Euclidean distance as a distance metric. Despite this, Wesolowski *et al*.^20^ found that Euclidean distance provided the most accurate prediction of travel frequency on a wide scale; however, this may have been due to the limited quality of road distance, travel cost and travel time data, or factors not accounted for, such as road quality. That said; road distance provided a better model fit for travel within rural areas^20^.

A strength of this analysis, by virtue of the data set, is that it allowed the quantification of movement patterns of key traveler groups – women traveling with children and youth workers. Children that women travel with are more likely to display clinical malaria incidence in high prevalence settings^21^, and qualitative research suggests that youth workers tend to travel for agricultural labor during the rainy season when malaria is most prevalent in the Sahel^32,33^. Further work could involve the determination of key drivers of individual travel, for instance, dependence of travel frequency on gender, age, socio-economic status and geographical covariates^34^. This may allow travel patterns to be more accurately extrapolated to other origins on the basis of their demography, with relevance to malaria transmission being determined based on a breakdown of malaria prevalence by age, sex and other characteristics. This is an important consideration for determining sources and sinks of malaria transmission. Further work could also explore the impact of changes in each country since these surveys were conducted. Population growth has been consistently occurring in all countries, alongside urbanization and road development, and in Mali there has been significant political instability and armed conflict, especially in the north of the country, since the surveys were finished there in March 2011.

Several weaknesses of the data set should be acknowledged. For instance, the survey sites were a judgment/convenience sample designed to capture a wide range of movement patterns, while taking advantage of existing relationships of local researchers with these communities. Since these sites were not chosen randomly, models fitted to these data are representative of the collection of study sites surveyed rather than of each country as a whole. While the fitted models may serve as proxies for each country, the selection of 3–5 survey sites by judgment/convenience will lead to biases in model parameter estimates and likelihood measures. For instance, in Tanzania, most of the survey sites were relatively urban, which could lead to a model and parameter values being favored that apply well for travel originating from larger population centers. In Burkina Faso, the survey sites were in or within the vicinity of the capital city, Ouagadougou, which could have favored a model and parameter values that accommodate capital city effects. In Zambia, several of the survey sites were border towns, and in Mali, the survey sites were relatively rural, aside from those in Bamako and Mopti, the urban-rural balance of which could have influenced the favored model and parameter estimates.

Additional biases could result from the nature of the survey. For instance, in the questionnaire, respondents were asked about their three most recent short-term and long-term trips (up to six in total). This could introduce a bias towards trips in the months preceding the interviews in two ways – recall bias, since recent trips may be easier to remember, and “trip clipping” since, for people who have taken many trips, only the recent ones will be recorded. Discrepancies for travel within the vicinity of a survey site may also have been introduced as travel within the commune/ward/city of origin was not considered travel in the survey, and there may have been some ambiguity over what constituted the commune/ward/city of origin. Furthermore, this travel criteria led to exclusion of short scale movements, the scale of exclusion for which varied depending on commune/ward/city size. A potential selection bias against frequent travelers may also have been present, as these travelers may have been traveling at the time of the survey. That said; there are biases in other data sources that survey data can help to elucidate – e.g. cell phone ownership and usage patterns that lead to biases in anonymous cell phone signal data^12^. Ultimately, a research agenda is needed that synergizes the strengths of multiple complementary data sets, elucidating each others’ biases and obtaining an accurate picture of human movement patterns^2^.

Both models captured most mobility trends qualitatively well; the gravity model provided the best fit to the data overall (Table 1), but neither the gravity nor radiation model was uniformly superior in its predictive ability. The gravity model tended to provide a better fit for travel to large populations (Figs 2 and 4), while the radiation model tended to provide a better fit for nearby populations (Figs 3 and 5). Similar trends were seen for the gravity model for women traveling with children and youth workers (Supplementary Figures 2 and 3), with youth workers being more attracted to large population centers, and women traveling with children being less attracted (Table 3). Future work could address modifying gravity and radiation models to achieve a more nuanced description of travel to nearby locations and mega-cities, and the drivers of individual travel, such as age, gender and socio-economic status. These spatial coupling models could then be linked to detailed transmission models of malaria and other diseases, to understand the role that human movement plays in their dynamics, and to better inform strategies to control their spread.

## Electronic supplementary material

Supplementary Information

Dataset 1
