# Supplementary material for: Mathematical models of human mobility of relevance to malaria transmission in Africa
Source: Sci Rep. 2018 May 16;8:7713. doi: 10.1038/s41598-018-26023-1 (PMC5955928; doi:10.1038/s41598-018-26023-1)
Supplement: Supplementary file 1 — Supplementary Information [file 41598_2018_26023_MOESM1_ESM.pdf]

Supplementary Material

Mathematical models of human mobility of relevance to malaria transmission in Africa

John M. Marshall[1,2*], Sean L. Wu[2], Hector M. Sanchez C.[2], Samson S. Kiware[3], Micky Ndhlovu[4], André Lin Ouédraogo[5,6], Mahamoudou B. Touré[7], Hugh J. Sturrock[8], Azra C. Ghani[1], Neil M. Ferguson[1]

[1]MRC Centre for Outbreak Analysis and Modelling, Department of Infectious Disease Epidemiology, Imperial College London, London, UK

[2]Divisions of Biostatistics and Epidemiology, School of Public Health, University of California, Berkeley, California, USA

[3]Environmental Health and Ecological Sciences Thematic Group, Ifakara Health Institute, Dar es Salaam, Tanzania

[4]Chainama College of Health Sciences, Lusaka, Zambia

[5]Centre National de Recherche et de Formation sur le Paludisme, Ouagadougou, Burkina Faso

[6]Institute for Disease Modeling, Bellevue, Washington, USA

[7]Malaria Research and Training Center, University of Bamako, Bamako, Mali

[8]Malaria Elimination Initiative, Global Health Group, University of California, San Francisco, California, USA

[*]Corresponding author

Corresponding author: John M. Marshall: john.marshall@berkeley.edu

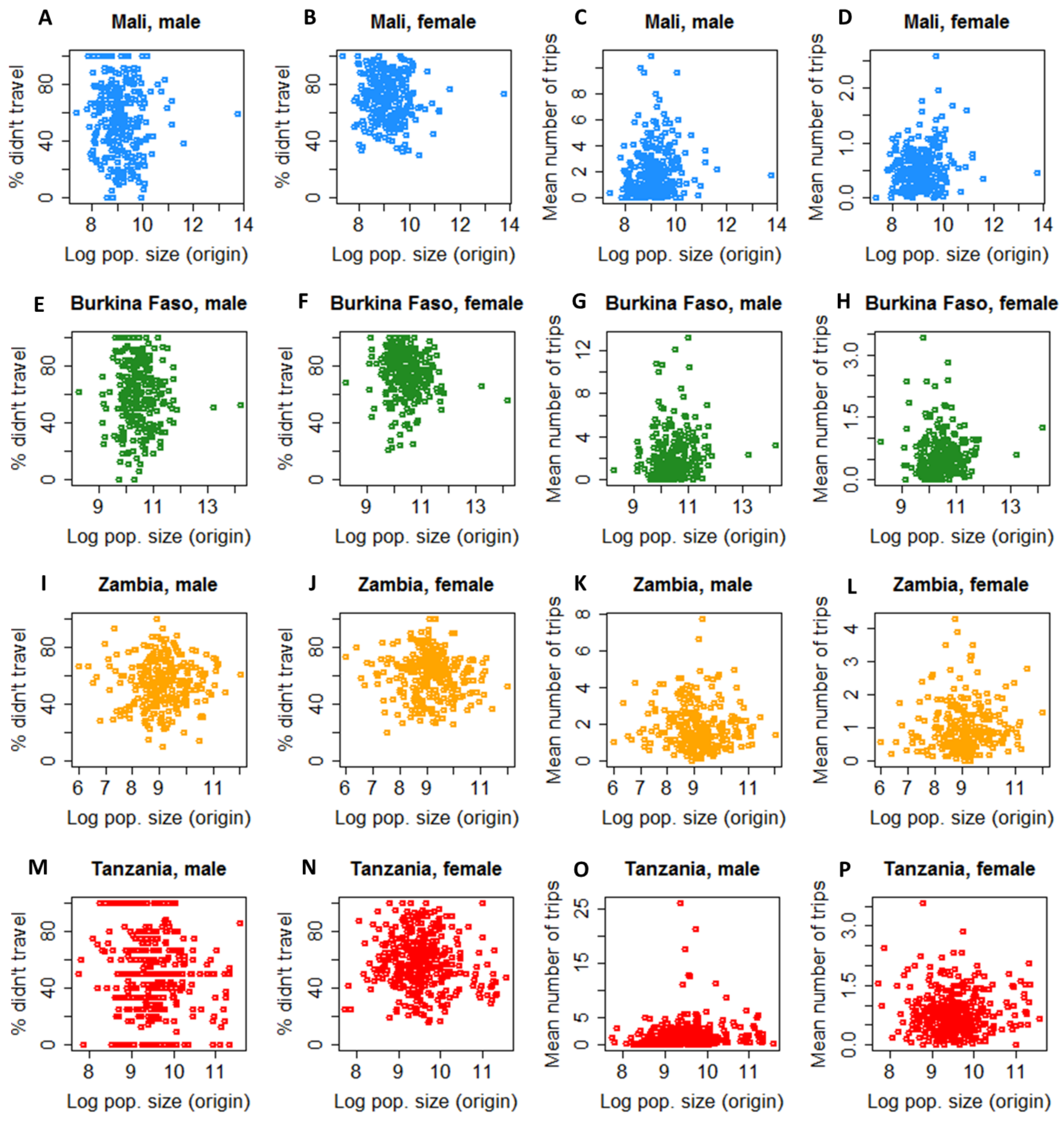

Supplementary Figure 1. Dependence of trip frequency on origin population size. Trip frequency measured by: i) the proportion of male and female interviewees who didn't travel in the last year; and ii) the mean number of trips made by male and female interviewees in the last year versus origin population size (log scale) for each of the four survey countries. Interestingly, there is no suggestion of a relationship between origin population size and travel frequency for either males or females.

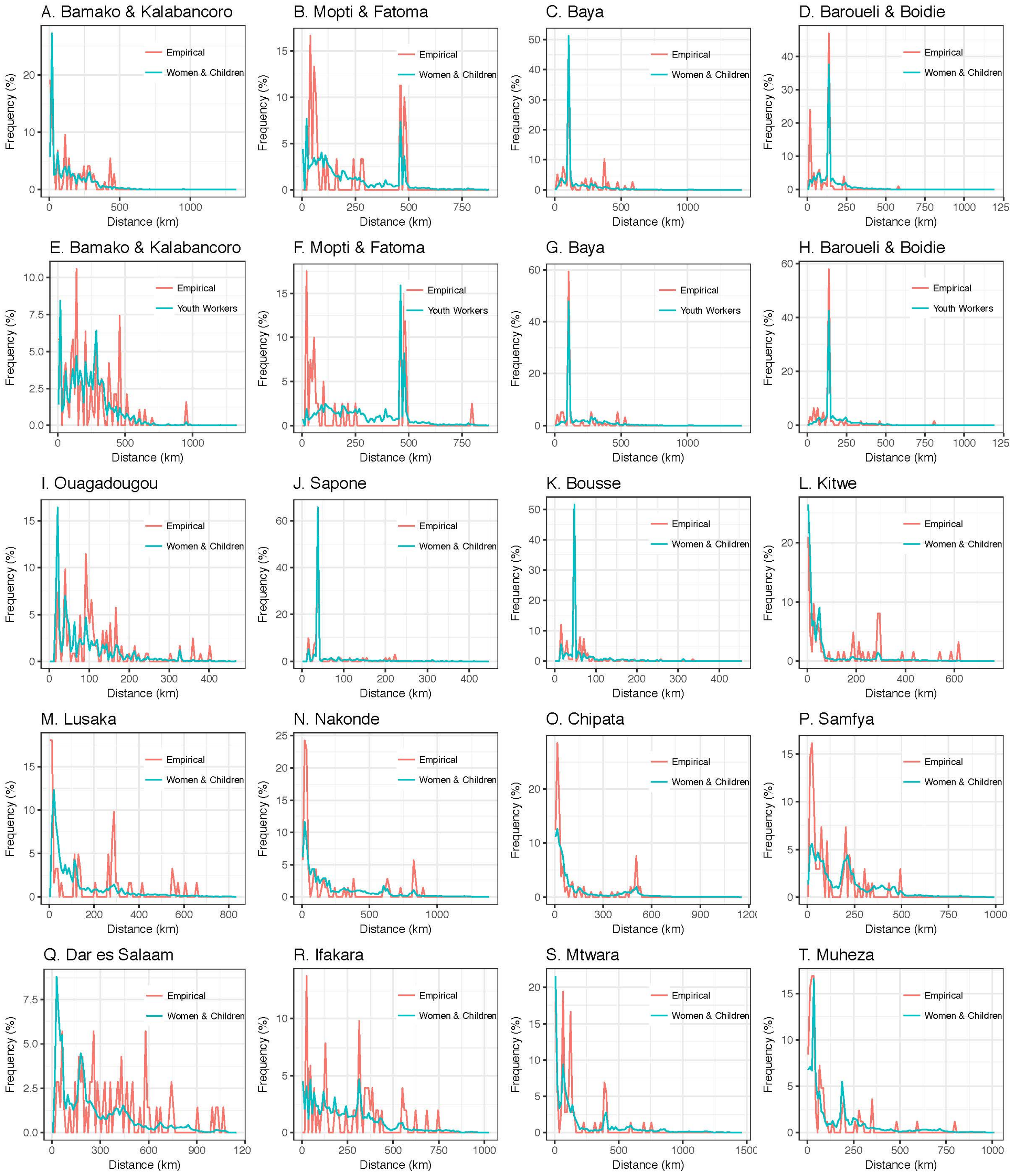

Supplementary Figure 2. Empirical and model-predicted distance distributions for gravity model fitted to individual countries with trips partitioned according to traveler group. Predicted travel frequencies are from gravity models fitted to travel data specific to each key traveler group with the destination population size raised to a power, τ, and parameter values in

Table 3. Distance distributions are shown for trips beginning at survey sites in Mali (panels A-H), Burkina Faso (panels I-K), Zambia (panels L-P) and Tanzania (panels Q-T). Trips are partitioned according to key traveler groups: (i) women traveling with children (for all survey countries), and (ii) youth workers (for Mali).

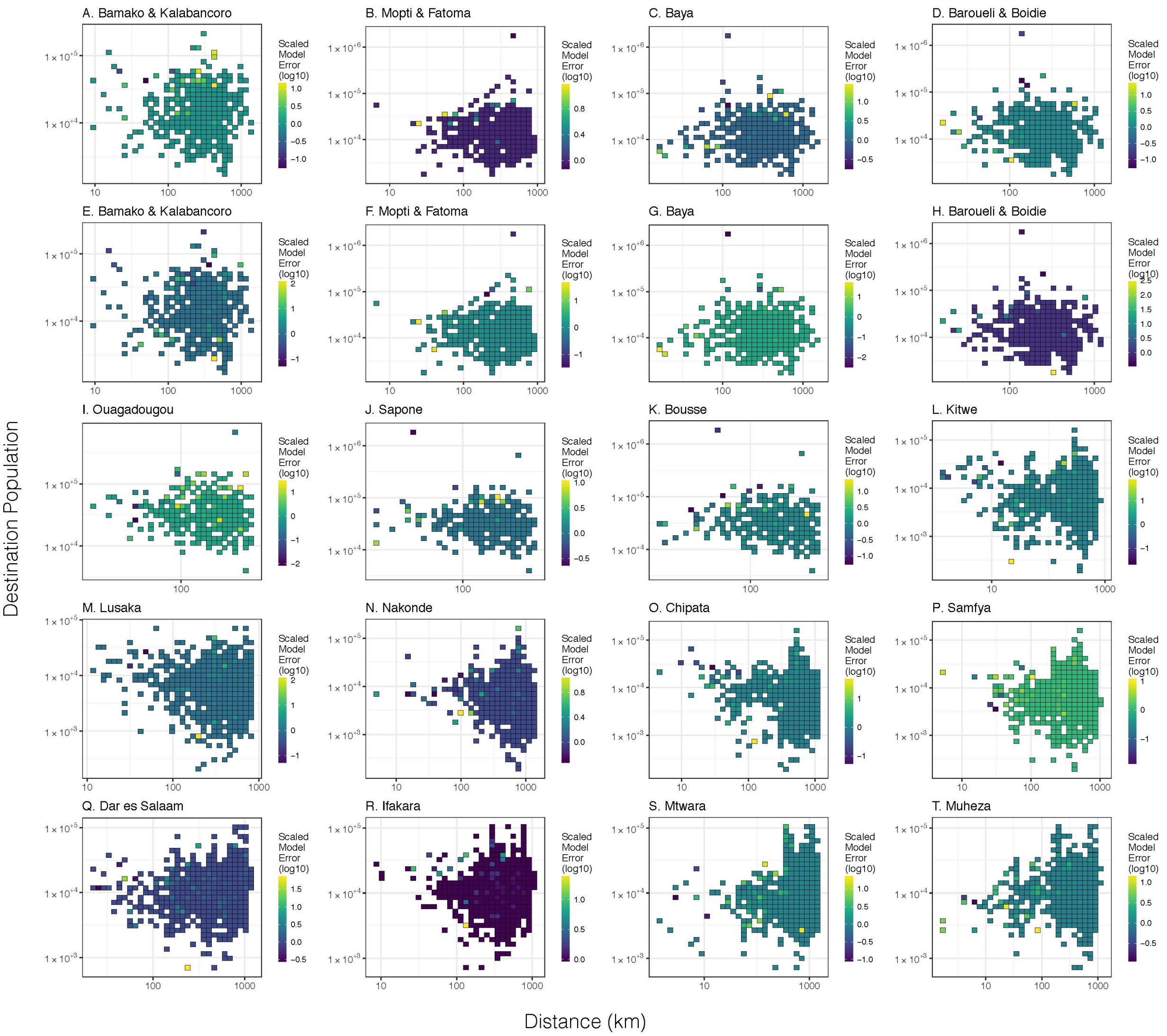

Supplementary Figure 3. Relative prediction error for gravity model fitted to individual countries with trips partitioned according to traveler group. Relative prediction error versus destination population size and trip distance for trips beginning at survey sites in Mali (panels A-H), Burkina Faso (panels I-K), Zambia (panels L-P) and Tanzania (panels Q-T). Trips are partitioned according to key traveler groups: (i) women traveling with children (for all survey countries), and (ii) youth workers (for Mali). Predicted travel frequencies are from gravity models fitted to travel data specific to each key traveler group with the destination population size raised to a power, τ, and parameter values in Table 3. Grid cells represent the average scaled model error for destinations falling within the corresponding range of destination population sizes and trip distances.

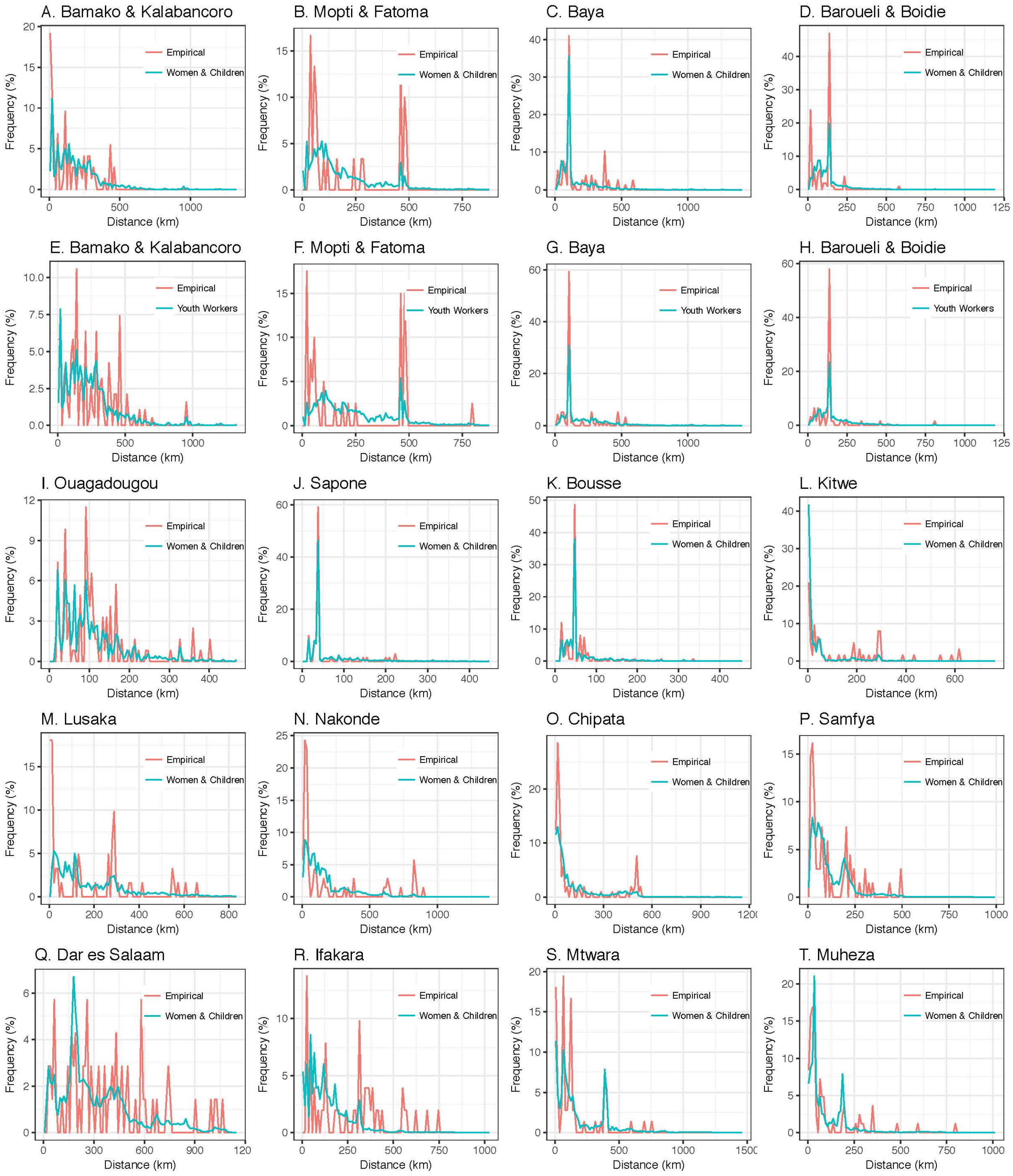

Supplementary Figure 4. Empirical and model-predicted distance distributions for radiation model fitted to individual countries with trips partitioned according to traveler group. Predicted travel frequencies are from radiation model B fitted to travel data specific to each key traveler group with parameter values in Table 3. Distance distributions are shown for

trips beginning at survey sites in Mali (panels A-H), Burkina Faso (panels I-K), Zambia (panels L-P) and Tanzania (panels Q-T). Trips are partitioned according to key traveler groups: (i) women traveling with children (for all survey countries), and (ii) youth workers (for Mali).

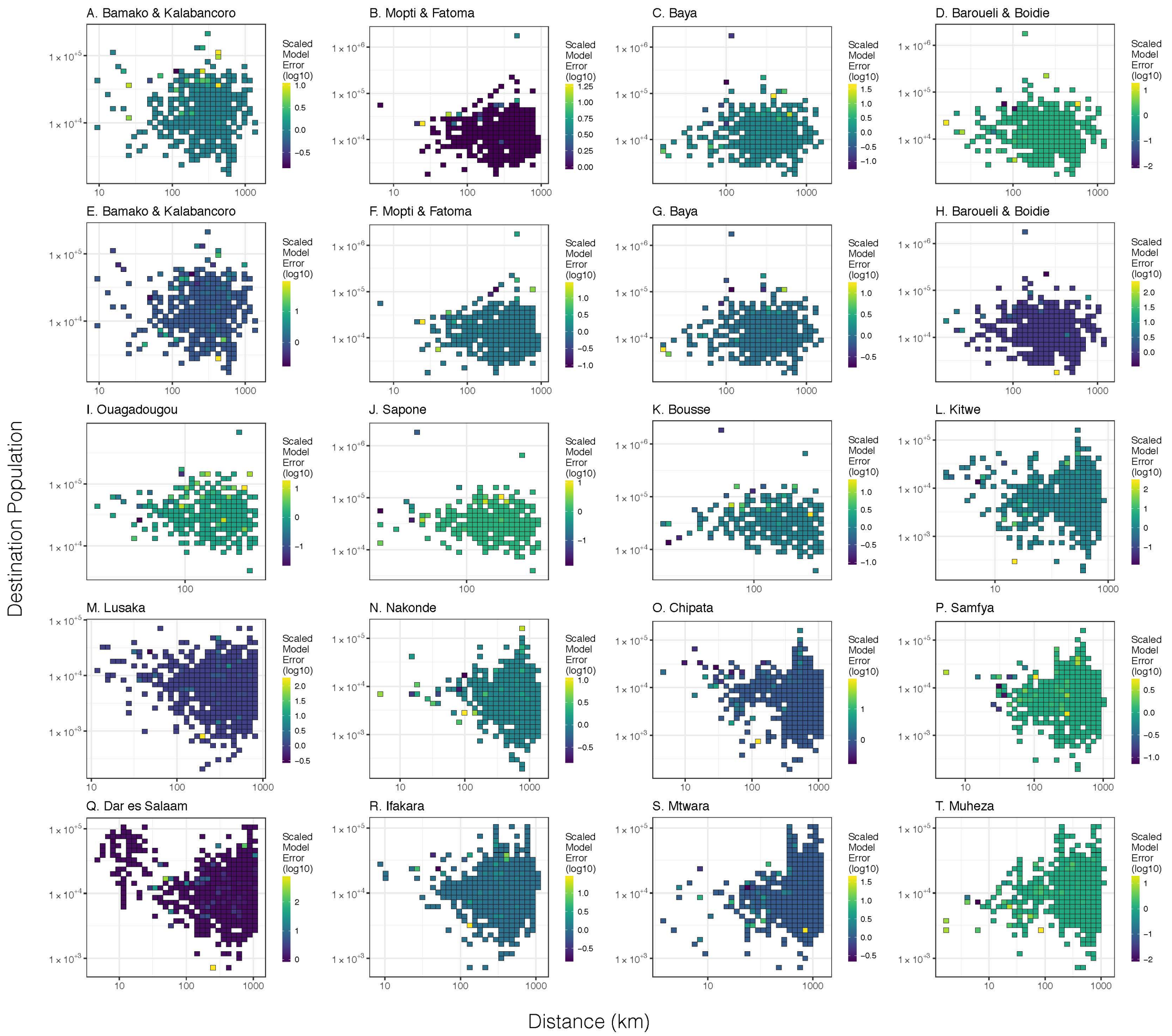

Supplementary Figure 5. Relative prediction error for radiation model fitted to individual countries with trips partitioned according to traveler group. Relative prediction error versus destination population size and trip distance for trips beginning at survey sites in Mali (panels A-H), Burkina Faso (panels I-K), Zambia (panels L-P) and Tanzania (panels Q-T). Trips are partitioned according to key traveler groups: (i) women traveling with children (for all survey countries), and (ii) youth workers (for Mali). Predicted travel frequencies are from radiation model B fitted to travel data specific to each key traveler group with parameter values in Table 3. Grid cells represent the average scaled model error for destinations falling within the corresponding range of destination population sizes and trip distances.

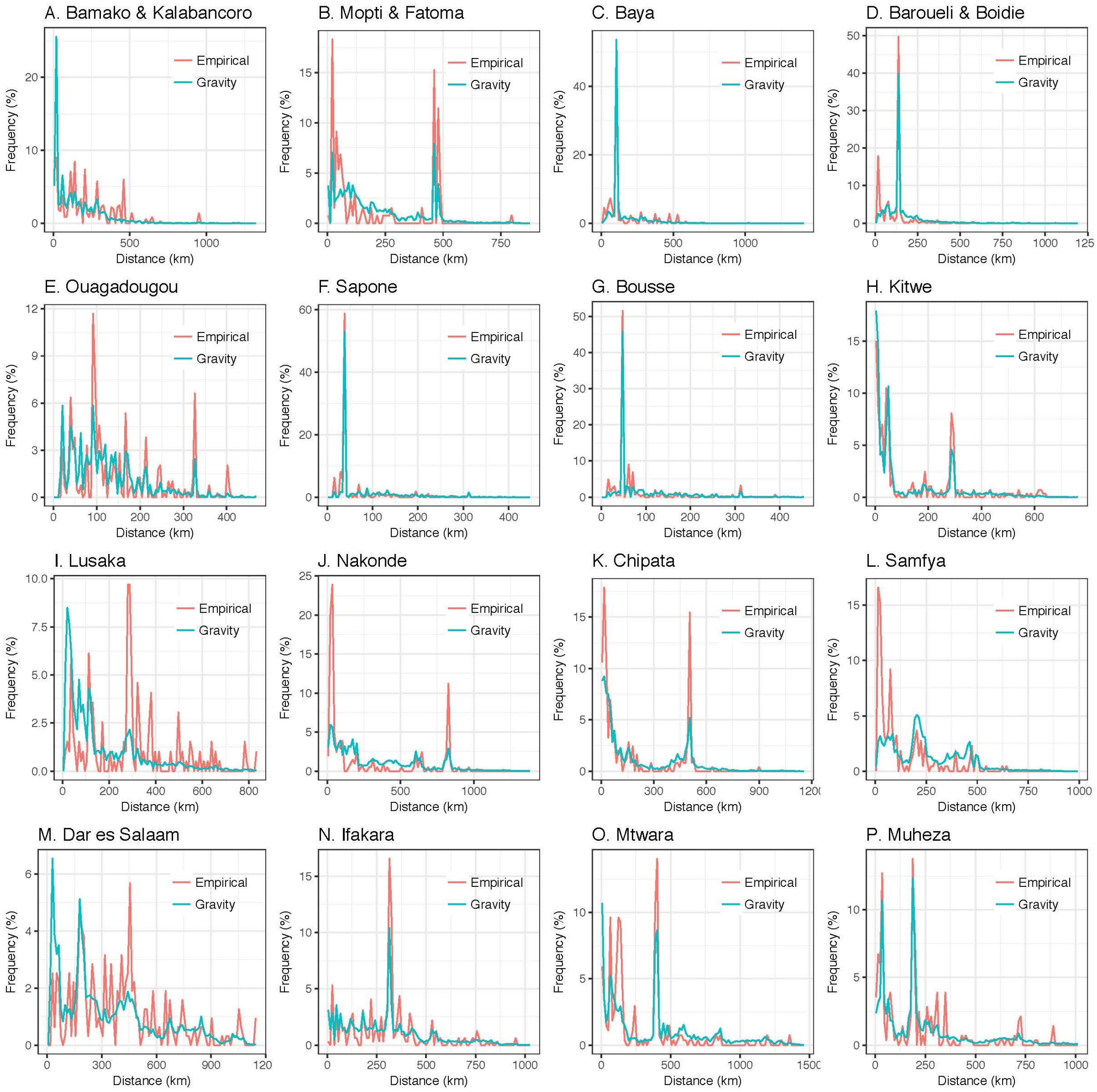

Supplementary Figure 6. Empirical and model-predicted distance distributions for gravity model fitted to all countries simultaneously. Predicted travel frequencies are from the gravity model with the destination population size raised to a power, τ, and parameter values in Table 1. Distance distributions are shown for trips beginning at survey sites in Mali (panels A-D), Burkina Faso (panels E-G), Zambia (panels H-L) and Tanzania (panels M-P).

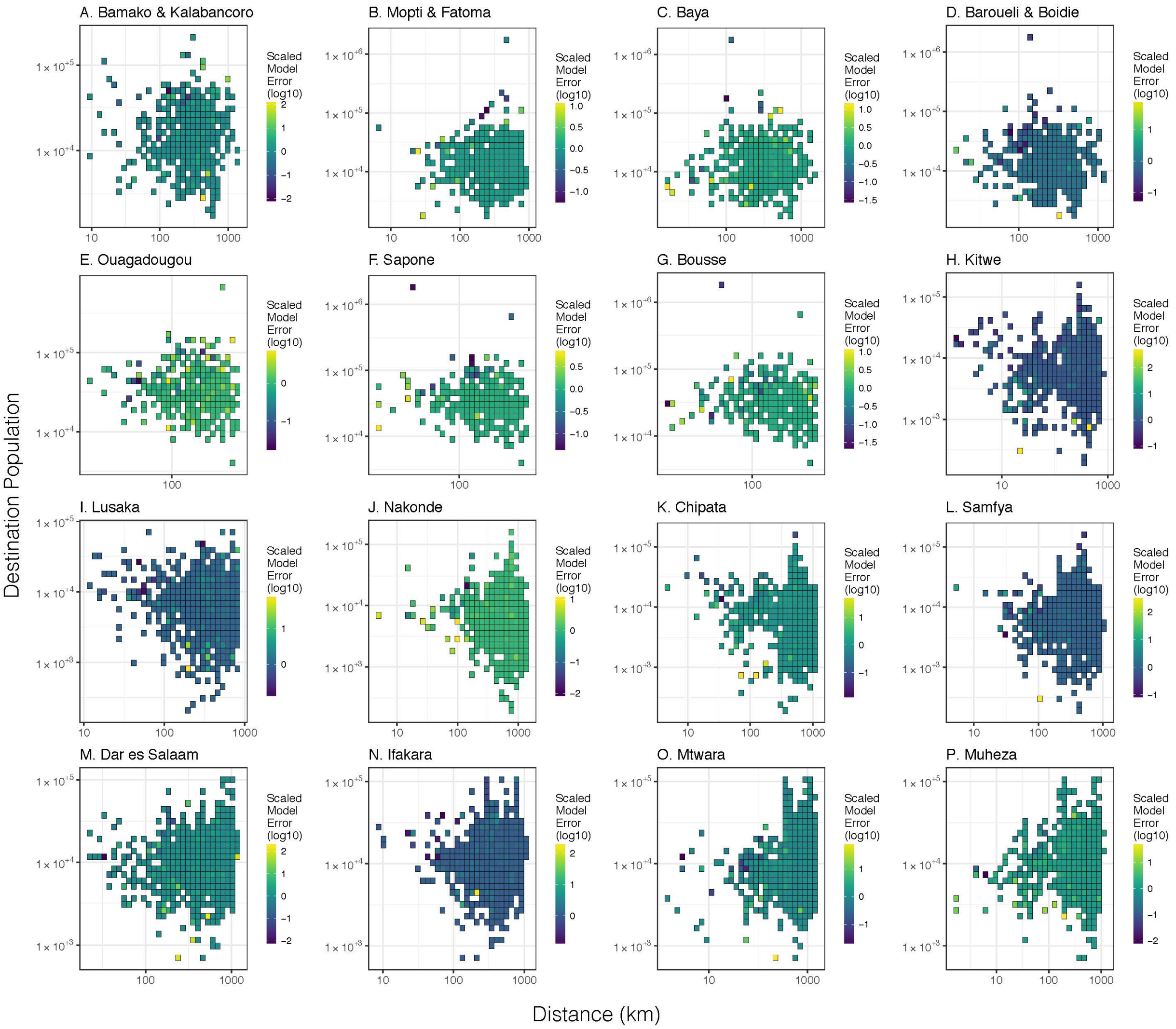

Supplementary Figure 7. Relative prediction error for gravity model fitted to all countries simultaneously. Relative prediction error versus destination population size and trip distance for trips beginning at survey sites in Mali (panels A-D), Burkina Faso (panels E-G), Zambia (panels H-L) and Tanzania (panels M-P). Predicted travel frequencies are from the gravity model applied to all countries simultaneously with the destination population size raised to a power, τ, and parameter values in Table 1 (all countries). Grid cells represent the average scaled model error for destinations falling within the corresponding range of destination population sizes and trip distances.

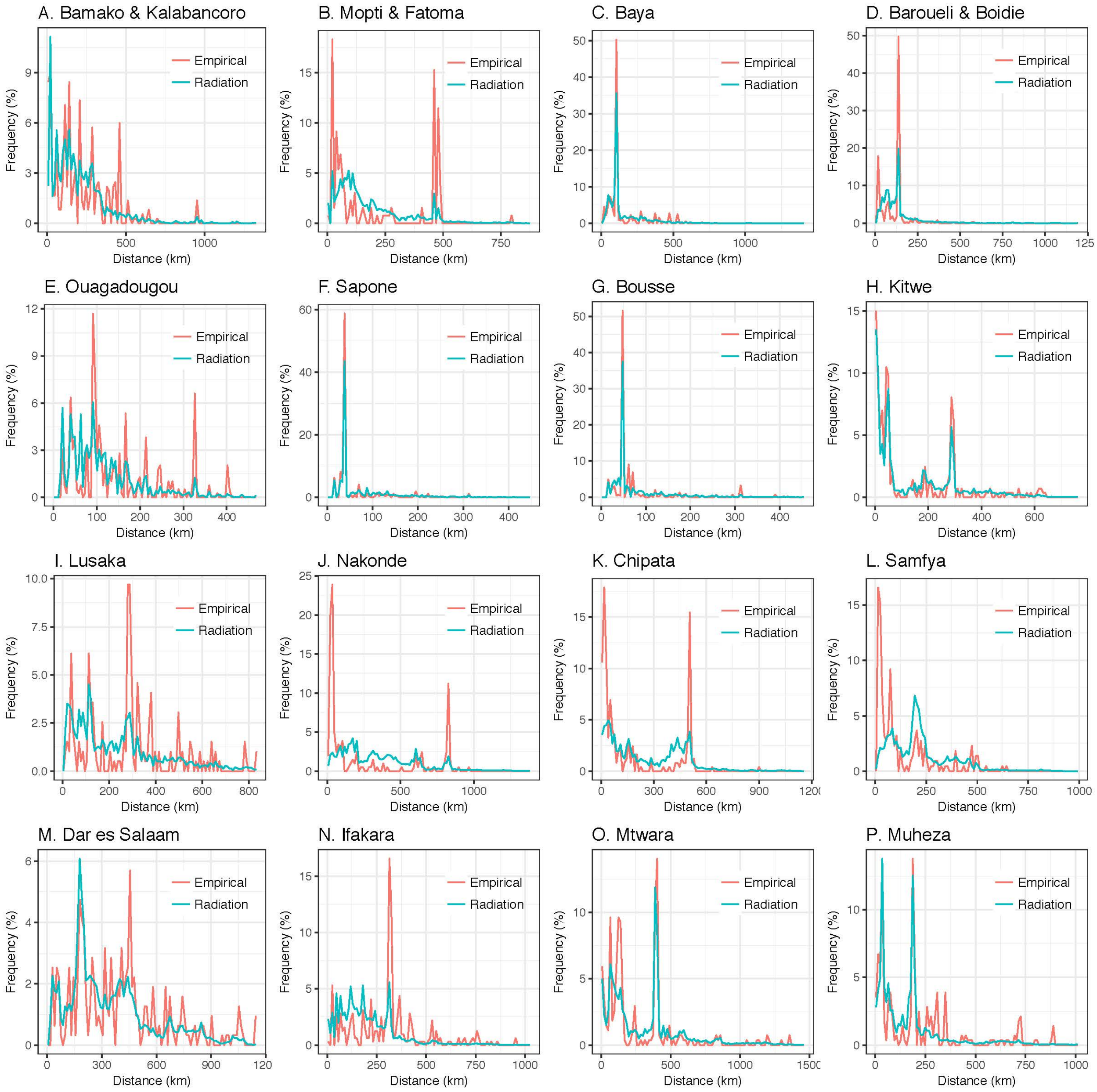

Supplementary Figure 8. Empirical and model-predicted distance distributions for radiation model fitted to all countries simultaneously. Predicted travel frequencies are from radiation model B with parameter values in Table 1. Distance distributions are shown for trips beginning at survey sites in Mali (panels A-D), Burkina Faso (panels E-G), Zambia (panels H-L) and Tanzania (panels M-P).

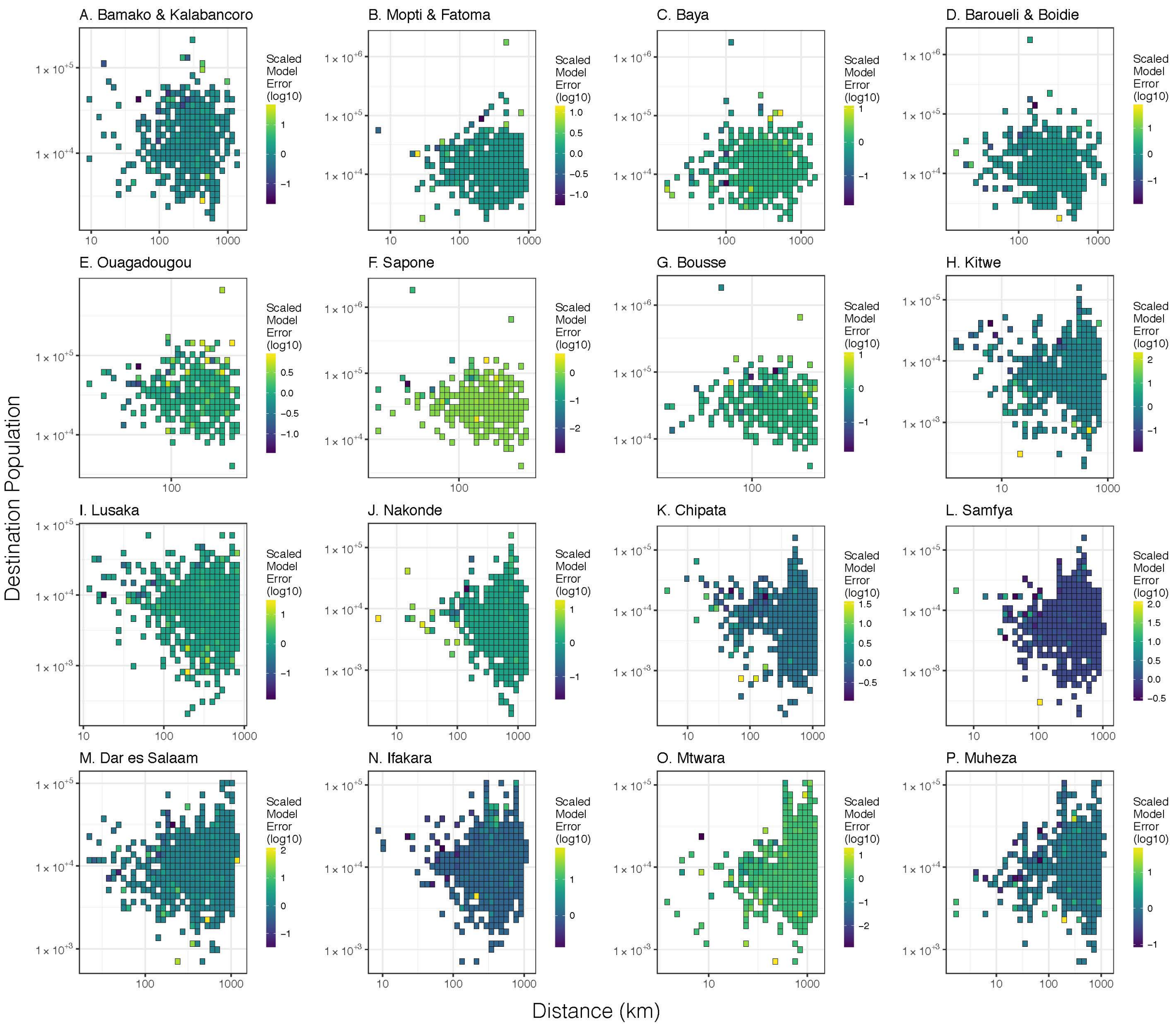

Supplementary Figure 9. Relative prediction error for radiation model fitted to all countries simultaneously Relative prediction error versus destination population size and trip distance for trips beginning at survey sites in Mali (panels A-D), Burkina Faso (panels E-G), Zambia (panels H-L) and Tanzania (panels M-P). Predicted travel frequencies are from radiation model B applied to all countries simultaneously with parameter values in Table 1 (all countries). Grid cells represent the average scaled model error for destinations falling within the corresponding range of destination population sizes and trip distances.

Supplementary File 1. Population and trip data for each survey country. Excel file containing two data sheets for each survey country – a data sheet containing a list of communes (for Mali and Burkina Faso) and wards (for Zambia and Tanzania), their corresponding higher administrative units, coordinates and population sizes, and another data sheet containing a list of origin and destination communes/wards, and a traveler group, as determined by the cluster analysis of Marshall *et al.* (1). Communes and wards are indexed with a unique ID in the former data sheet for each country.

References:

1. Marshall, J. M. *et al.* Key traveler groups of relevance to spatial malaria transmission: A survey of movement patterns in four sub-Saharan African countries. *Malar. J.* **15**, 200 (2016).
